# Dynamics of SARS-CoV-2 Major Genetic Lineages in Moscow in the Context of Vaccine Prophylaxis

**DOI:** 10.3390/ijms232314670

**Published:** 2022-11-24

**Authors:** Vladimir A. Gushchin, Andrei A. Pochtovyi, Daria D. Kustova, Darya A. Ogarkova, Ivan Y. Tarnovetskii, Elizaveta D. Belyaeva, Elizaveta V. Divisenko, Lyudmila A. Vasilchenko, Elena V. Shidlovskaya, Nadezhda A. Kuznetsova, Artem P. Tkachuk, Egor A. Slutskiy, Gleb I. Speshilov, Andrei G. Komarov, Alexander N. Tsibin, Vladimir I. Zlobin, Denis Y. Logunov, Alexander L. Gintsburg

**Affiliations:** 1 Federal State Budget Institution “National Research Centre for Epidemiology and Microbiology Named after Honorary Academician N. F. Gamaleya” of the Ministry of Health of the Russian Federation, 123098 Moscow, Russia; 2 Department of Virology, Biological Faculty, Lomonosov Moscow State University, 119991 Moscow, Russia; 3 Moscow Healthcare Department, 127006 Moscow, Russia; 4 Department of Infectiology and Virology, Federal State Autonomous Educational Institution of Higher Education I.M. Sechenov, First Moscow State Medical University of the Ministry of Health of the Russian Federation (Sechenov University), 119435 Moscow, Russia

**Keywords:** SARS-CoV-2, vaccine prophylaxis, epidemiology, dynamics of genetic lines, reproduction number

## Abstract

Findings collected over two and a half years of the COVID-19 pandemic demonstrated that the level immunity resulting from vaccination and infection is insufficient to stop the circulation of new genetic variants. The short-term decline in morbidity was followed by a steady increase. The early identification of new genetic lineages that will require vaccine adaptation in the future is an important research target. In this study, we summarised data on the variability of genetic line composition throughout the COVID-19 pandemic in Moscow, Russia, and evaluated the virological and epidemiological features of dominant variants in the context of selected vaccine prophylaxes. The prevalence of the Omicron variant highlighted the low effectiveness of the existing immune layer in preventing infection, which points to the necessity of optimising the antigens used in vaccines in Moscow. Logistic growth curves showing the rate at which the new variant displaces the previously dominant variants may serve as early indicators for selecting candidates for updated vaccines, along with estimates of efficacy, reduced viral neutralising activity against the new strains, and viral load in previously vaccinated patients.

## 1. Introduction

The emergence of the SARS-CoV-2 virus in the human population caused a global COVID-19 pandemic [1,2]. The lack of deterrents, such as pre-existing level immunity, contributed to its rapid spread and high infectivity in the first phase. The most significant outcome of the first year of the pandemic was the commercialisation of highly effective vaccines [3,4,5,6]. All major prophylactics were derived from modern approaches involving targeted antigen gene delivery using adenoviral vectors [5,6] or lipid nanoparticles containing prepared mRNA [3,4]. In all cases, the only immunogen was the S glycoprotein of the Wuhan variant. Immunisation with mRNA and adenoviruses leads to the formation of effective virus-neutralising humoral and cellular responses. The efficacy of the main vaccines against the initial strains was >90%, which facilitated rapid and effective mass vaccination. The reproductive number, Rt, was calculated to determine the necessity of establishing a vaccination level of at least 76% to facilitate the natural termination of viral circulation [7].

The emergence and widespread distribution of new variants of WHO concern significantly changed the epidemiological situation [8]. All variants of concern carried mutations in the receptor-binding domain of the S protein. Mutations are the primary mechanism by which the virus maintains intense transmissibility in the face of growing level immunity. The new variants demonstrated greater transmissibility, which is likely due to higher viral load, lower required viral load for infection, and continued infectivity among immunised populations [9,10]. The Delta variant (B.1.617.2 + AY.X) was shown to be 40–60% more transmissible than the Alpha variant (B.1.1.7 + Q.X) [11,12]. In a UK study, the Delta variant was found to be 40–80% more transmissible than the Alpha variant [10]. In non-contact transmission simulations using Syrian hamsters, Omicron demonstrated the same or higher transmission rates than those of Delta [13]. Researchers in Denmark analysed the transmission dynamics of Omicron and Delta variants within families and determined that Omicron was about 2.6 times more contagious than the Delta variant among vaccinated and revaccinated individuals, whereas among unvaccinated people, Omicron was only 1.17 times more contagious than Delta [14,15]. These studies were conducted in countries that used vaccines developed by Pfizer, Moderna, and AstraZeneca as the main drugs. Data on the virological and epidemiological features of the variants of concern in human populations vaccinated with Sputnik V and Lite are not currently available.

Various factors may influence the rate of spread of specific genetic lineages, including both the genetic characteristics of the virus itself and the characteristics of the population, such as the vaccination drugs used, the integral level of level immunity, and non-pharmacological measures of prevention [10]. This study focused on SARS-CoV-2 immunity in response to emerging variants in Moscow, the largest city in the Russian Federation. The main drugs employed for managing COVID-19 in Moscow are Sputnik V [6] and Sputnik Light [16], which were formulated using 26 serotypes and 5 adenoviruses as delivery vehicles. It is worth noting that these vaccine preparations were approved in 71 countries with a population of 4 billion people [17]. They are widespread in Russia, Argentina, and San Marino.

We analysed the dynamics of the composition of the main SARS-CoV-2 genetic lineages throughout the pandemic, evaluated the dynamic parameters of the spread rate of new variants, analysed the probable factors contributing to their spread, and assessed the effectiveness of Sputnik V and Sputnik Lite drugs in Moscow during the periods in which the main genetic variants spread.

## 2. Results

### 2.1. Dynamics of SARS-CoV-2 Major Genetic Lineages in Moscow, COVID-19 Incidence, and Level Immunity

**Figure 1:**
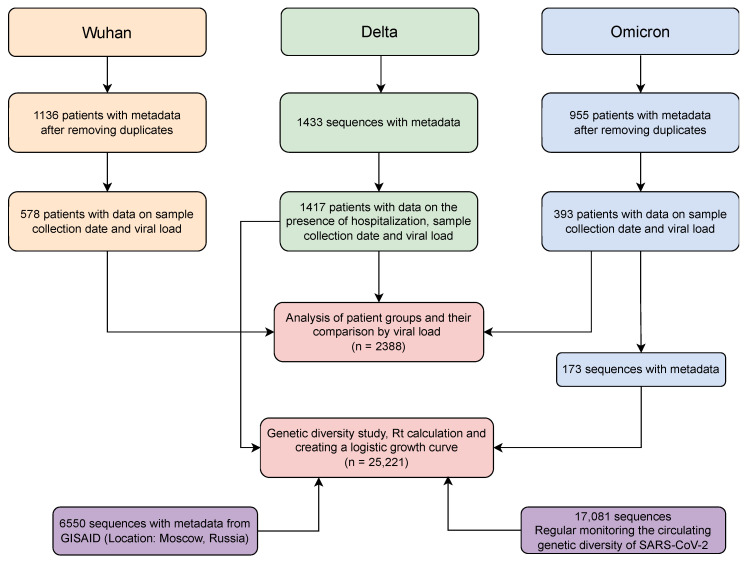
Database in preparation for the analysis.

To obtain data on the dynamics of the main genetic lines, we performed genetic monitoring of SARS-CoV-2 variability in Moscow in different volunteer cohorts (initially infected and vaccinated, Figure 1).

**Figure 2:**
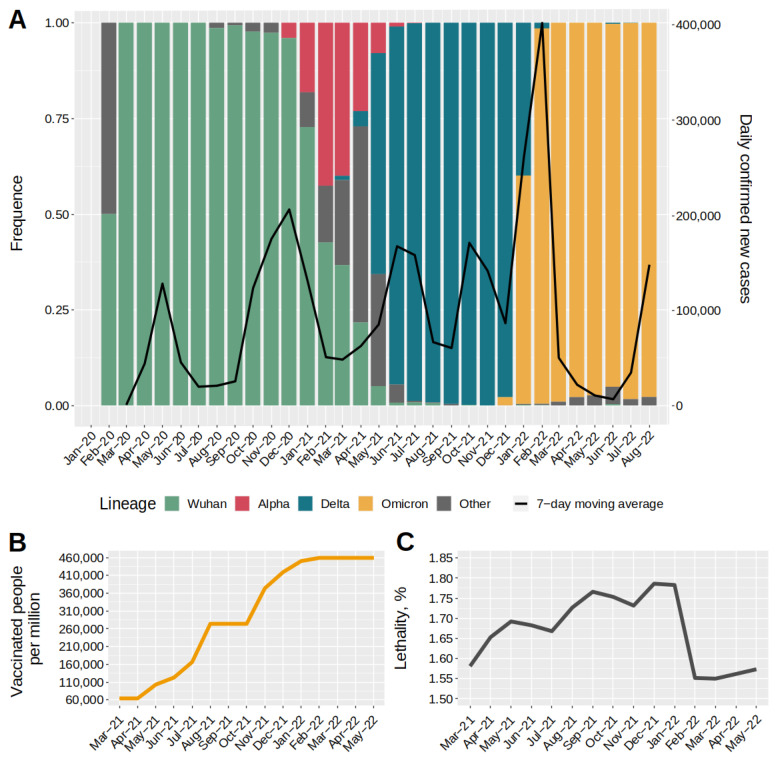
Dynamics of major SARS-CoV-2 genetic lineages in Moscow, COVID-19 incidence, and number of vaccinated individuals. (**A**) Profile of changes in major genetic lineages since viral outbreak in Moscow. The second ordinate scale shows the number of new cases. The black line shows the dynamics of new cases. (**B**) Cumulative value of fully vaccinated patients during mass vaccination in 2021–2022. The ordinate axis shows the number of fully vaccinated people per 1 million inhabitants. (**C**) Value of lethality in 2021–2022. Lethality is expressed as a percentage and was calculated as the ratio of patients who died to those who became ill.

Monitoring allowed us to collect data on the structure of the complete SARS-CoV-2 genome, which were combined with other publicly available data and used to construct a diagram reflecting the structure of the main genetic variants (Figure 2). The combined database included information on 25,221 genomes, for which a genetic lineage was identified. In summary, we identified the main variants, including Wuhan (B.1.X + B.1.1.X), Alpha (B.1.1.7 + Q.X), Delta (B.1.617.2 + AY.X), and Omicron (B.1.1.529 + BA.X). A more detailed breakdown of strain composition in the main waves is provided in the Supplementary Material (Figure S1). Together with the variability data, we used the COVID-19 incidence information (black line) from the COVID-19 open registry [18] and materials on the dynamics of level immunity formation (Figure 2B) [19].

The dominant lineages from early 2020 to December 2020 were B.1 and its daughter variants (B.1.X, hereafter the Wuhan group), which accounted for nearly 100% of lineage diversity. In late December 2020, the first cases of infection with the Alpha variant were observed. The emergence of Alpha led to a partial displacement of the B.1 + B.1.X lines, reducing their proportion to ~53%. February–March 2021 were characterised by a rapid increase in the proportion of variant Delta (B.1.617.2 + AY.X), which displaced all other circulating lines by November 2021. A similar pattern was observed for Omicron, which displaced all circulating variants and became the dominant variant in January 2022, when it accounted for more than 73% of infections.

Analysis of the data on the number of detected COVID-19 cases allowed us to distinguish five completed waves of disease growth and a sixth wave beginning at the time of writing. The first two waves were caused by the original viral variants (B.1.X + B.1.1.X) and occurred in the spring and fall–winter periods of 2020. These peaks were separated by summer months (July–September 2020). The next two waves in Moscow caused by the Delta variant (B.1.617.2 + AY.X) occurred in the spring–summer and fall periods of 2021. These waves were also separated by the summer months (August–September 2020), during which there was a decrease in incidence. The third and fourth waves of incidence, during which the Delta variant dominated (Figure 2A), were characterised by a reduced intensity compared to the previous two. Further, the greatest increase in incidence over the whole pandemic period was observed at the end of 2021 with a peak in February 2022. The fifth wave completely coincided with the wide spread of the Omicron variants BA.1.X and BA.2, whereas the sixth wave was caused by the spread of BA.5.X variants. Analysing the composition of genetic lineages in the context of morbidity revealed that in four of the six cases, an increase in morbidity was observed against the background of a change in the dominant genetic lineage of the virus. Only the first two and subsequent two waves were caused by the Wuhan and Delta variants. During this period, the level of level immunity created by vaccination cannot be considered sufficient [20]. Thus, the initial mass vaccination with Sputnik V and with Sputnik Light around mid-2021 was initiated after the second wave began. The vaccination campaign peaked in June 2021 and coincided with the third wave caused by the Delta variant. During this period, an average of over 75,000 people were vaccinated per week. The second peak of vaccination and revaccination was observed in the fall of 2021 and coincided with the fourth wave, caused by the continued circulation of the Delta variant. In total, of the 12 million Moscow residents, approximately half of the adult population was vaccinated during the entire vaccination period. This means that throughout the entire study period, the level of level immunity formed by vaccination against COVID-19 in Moscow did not reach the necessary level, and thus could not fully contain the spread of the virus in the population.

### 2.2. Dynamics of Circulating Variant Displacement by Primary SARS-CoV-2 Genetic Lineages

**Figure 3:**
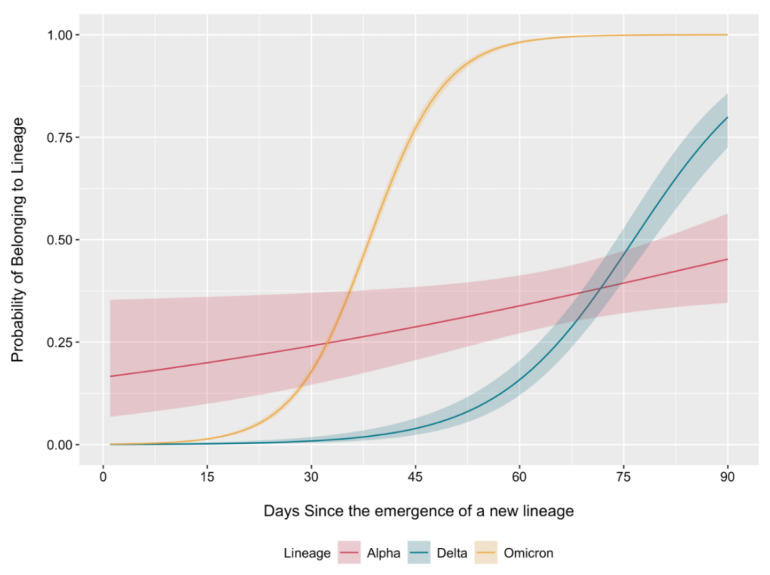
Binomial logistic regression for predicting the probability of sequenced sequences belonging to the variant of interest. The analysis was restricted to the first 90 days from the occurrence of each variant. Lines are plotted with 95% confidence intervals.

The obtained data on the changes in the composition of circulating genetic lines allowed us to conduct a comparative analysis of their logistic growth rates (Figure 3) following the approach proposed by Earnest et al. [10]. For this purpose, we assigned all genetic lines to larger genetic variants depending on the parental lineage. We considered lineage appearances at different times to facilitate an accurate comparison and considered the first 90 days after initial detection, which allowed us to estimate the logistic growth rate of dominant variants during the corresponding periods of appearance.

The logistic growth curves for Omicron and Delta had a more specific S-shape. The Omicron variant took approximately 37 days less than the Delta variant (38 days vs. 75 days) to reach a spread of over 50%. Alpha was characterised by a gentler increase in the proportion of the genetic landscape. Simultaneously, the probability of new sequences belonging to the Alpha variant at the start of its spread was 16.6%. This can be explained by the fact that this variant was circulating for some time before detection, which can most likely be explained by the fact that insufficient sequences were obtained for Moscow during this period. The logistic growth rate for Omicron was 1.80-fold and 11.49-fold higher than that for Delta and Alpha, respectively, and 6.38-fold higher for Delta variants relative to Alpha. The BA.5.2 genetic lineage accounting for the increased incidence in Moscow probably has a gentler logistic growth curve, which may be explained by difficulties in overcoming the immunity formed by the wide circulation of BA.1 and BA.2 variants in the last seven months against the background of the vaccination campaign.

### 2.3. Effective Reproduction Numbers of Major SARS-CoV-2 Genetic Lineages

To study the epidemiological features of the main genetic variants circulating in Moscow, we calculated their effective Rt. This index makes it possible to estimate how many people an infected patient can infect, on average, during the course of the disease. To calculate Rt, we used the genetic monitoring data and recorded the number of new cases. This allowed us to evaluate the contribution of each genetic variant in the epidemiological process.

**Figure 4:**
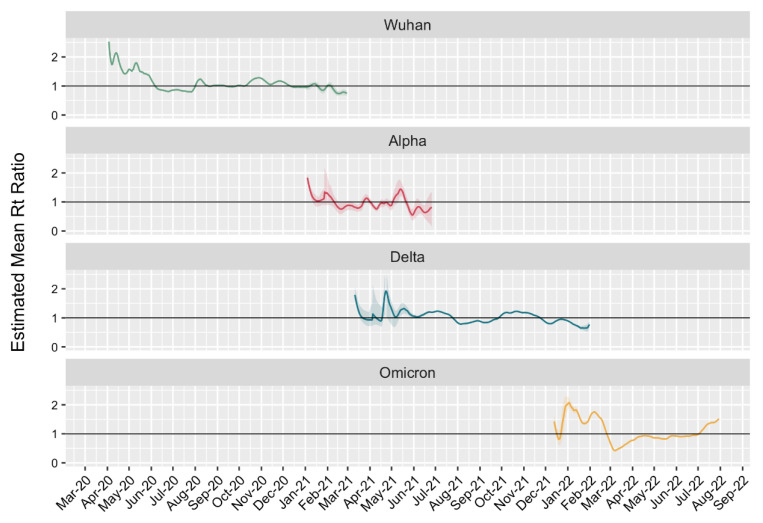
Change dynamics of Rt for main SARS-CoV-2 genetic lineages.

At the beginning of the pandemic, when the initial variants were spreading, Rt reached a maximum of 2.52 (95% CI 2.52–2.53). Thereafter, a marked decrease was observed, reaching <1 in May 2020 (Figure 4). The effective Rt was 1.83 (95% CI 1.71–1.99) at the beginning of the prevalence period for Alpha. Under the conditions of the first incidence peak, that for Delta was 1.79 (95% CI 1.71–2.07), which decreased to 1.21 during the second peak. A different pattern was observed for the Omicron variants. At the very beginning of their spread, a low Rt was observed, which was associated with the receipt of samples mainly from passengers arriving from abroad and placed immediately in quarantine. However, these individuals had no opportunity to spread the virus. The main increase in the number of infected persons and the spread of the virus was observed from mid-December 2021 to late January 2022, when the Rt value exceeded 1 for 47 days. This estimate shows that at the time of entry, the new variant has a significant epidemiological advantage, leading to fairly rapid displacement of precursor variants.

We calculated the average Rt ratio of co-circulating lines to estimate their transmissibility. The Alpha:Wuhan ratio was 1.09, Delta:Alpha ratio was 1.57, and Omicron:Delta ratio was 1.75. This establishes that Alpha, Delta, and Omicron in Moscow were on average 9%, 57%, and 75% more transmissible, respectively, relative to their predecessors.

### 2.4. Viral Load and Mutation Composition of the S Protein in the Main Genetic Lineages of SARS-CoV-2

**Figure 5:**
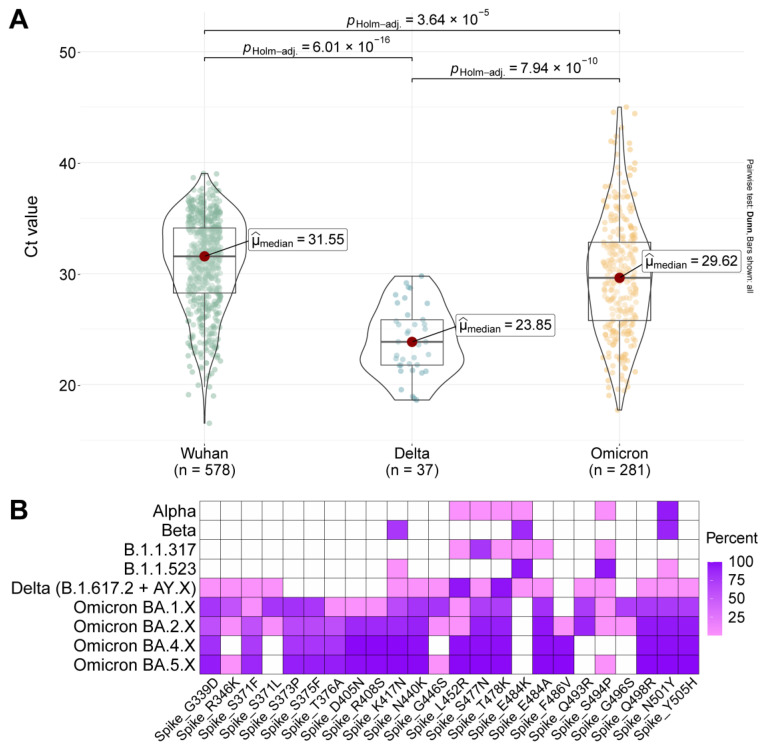
Viral load and mutation composition in the S protein of major SARS-CoV-2 genetic lineages. (**A**) PCR threshold cycle in nasopharyngeal swab compositions in primary patients during Wuhan-, Delta-, and Omicron-dominant periods. The differences between Delta and Wuhan (*p* < 0.0001) and Delta and Omicron (*p* < 0.0001) were statistically significant. (**B**) Composition of mutations within the RBD of the main genetic variants during Alpha-, Beta-, Delta-, Omicron-dominant periods and endemic variants SARS-CoV-2 in Moscow. The presence of amino acid substitutions are given relative to the Wuhan reference strain.

To assess the virological features responsible for new variants displacing previous variants, we examined the threshold cycle value for the main variants and the composition of mutations in the receptor binding domain (RBD) (Figure 5) and S protein (Figure S3).

Viral load is an important factor that contributes to viral transmission. The more viable the virus is upon entry, the higher its transmission efficiency. To calculate the threshold cycle, we used data from both outpatients and inpatients during different periods of dominance of Wuhan, Delta, and Omicron variants (Figure 5A). For outpatients, the median threshold cycles were 31.55 for Wuhan, 23.85 for Delta, and 29.62 for Omicron, indicating that the Delta variant released two orders of magnitude more viral load into the environment than the Wuhan and Omicron variants.

The transmission efficiency of the Delta and Omicron variants was most likely related to the composition of mutations in the RBD and S-protein (Figure 5B and Figure S3). Thus, the number of mutations in RBD in Omicron variants BA.4.X and BA.5.X has reached 17, and these new variants are characterized by the same L452R and T478K mutations as the Delta variant (Figure 5B and Figure S3). It is worth noting that variants BA.5.X (BQ.X, CL.1, BW.1) have now emerged with about 19 major mutations in RBD.

### 2.5. Effect of Vaccination on Viral Load Reduction

Effective vaccine prophylaxis does not guarantee that a vaccinated individual will not become ill. However, to reduce the risk of transmission, it is important that a vaccinated person who became sick does not become a source of infection. The viral load in vaccinated patients may be an indicator of a decreased likelihood of transmission in the population. We performed a viral load study in vaccinated individuals during the spread of the Delta and Omicron variants. To investigate the threshold cycle, we evaluated the viral load in a group of outpatients who were previously unvaccinated and vaccinated for COVID-19.

**Figure 6:**
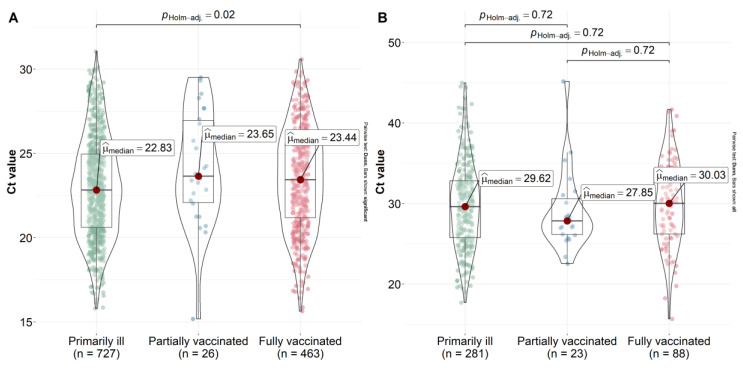
Ct value in primary detected, partially, and fully vaccinated patients in (**A**) the period that the Delta variant dominated and (**B**) the period that the Omicron variant dominated. The ordinate scale is represented by the Ct value.

The vast majority of the study samples collected during the spread of the Delta variant were from primary COVID-19 patients (*n* = 727). For partially (*n* = 26) and fully vaccinated (*n* = 463) patients, we collected enough samples to calculate statistical differences. The lowest viral load values were observed among fully and partially vaccinated outpatients (Figure 6). The lowest Ct value was observed in the primary disease group, with a median value of 22.83. Statistically significant differences were observed between fully vaccinated and initially sick patients (*p* = 0.02). This indirectly indicated the efficacy of vaccination with Sputnik V and Sputnik Lite against the Delta variant. When the Omicron variant dominated, no statistically significant differences in viral load were found between vaccinated, partially vaccinated, and primary disease groups (*p* = 0.72). In the fully vaccinated group (>21 days elapsed since the second dose), the viral load was comparable to that of the primary disease patients. This indicated a decrease in vaccine efficacy with respect to the Omicron variant.

## 3. Discussion

The continuation of the COVID-19 pandemic is clearly facilitated by the emergence and spread of new SARS-CoV-2 genetic variants that change the dynamics of the epidemiological process and elude the protection provided by vaccination and prior disease [21,22]. Even in countries with very high vaccination rates that show efficacy rates above 90%, new waves of disease are present. The need to update the composition of the vaccine strains was widely discussed. The WHO stated the urgent need to accumulate clinical data on the efficacy of Omicron S protein-based vaccines [23]. Key vaccine manufacturers announced the imminent market clearance of Omicron-modified variants [24,25,26]. The accumulated data during the pandemic regarding the variability of the virus, the vaccination used, and the dynamics of the epidemiological process should help accelerate the identification of potentially dangerous virus variants against which vaccine updates are needed. In this regard, one primary question emerged: what early indicators can be used to identify variants against which a vaccine update is necessary?

In most countries, the Delta variant and its daughter variants appeared in mid-2021, displacing previous variants, including Alpha. The logistical growth rate for Delta variants was 4.57 times that of Alpha. In a similar study conducted in New England, USA, these values were in the 1.37–2.63 range [10]. The logistic growth rates for Omicron were 1.81 and 8.29 times higher for Delta and Alpha, respectively, and 4.57 times higher for Delta relative to Alpha. Level immunity cannot be said to exist during the spread of the Delta variant, since the peak of vaccination with Sputnik V and Sputnik Lite occurred during the spread of the Delta variant; however, by the time the Omicron variant appeared, more than half of Moscow residents were vaccinated.

Logistic growth curve analysis indicated that the form of the logistic growth curve and the rate at which the previously circulating variants are displaced on the background of increasing morbidity can be used to identify potentially dangerous variants against which vaccine development is necessary. Thus, the shape of the curve during the period of pervasion of the Omicron variant was the most S-figurative. Logistic growth curve shape analysis can be performed on the data from any region to facilitate the identification of potentially dangerous variants in advance. Clearly, there may be differences from country to country caused, among other things, by immunological features of the population, but this will allow early vigilance against new variants. To ensure the accuracy of interpretations, it is necessary to collect data on level immunity and its intensity.

The emergence of variants of concern in Moscow led to a peak increase in the effective Rt number. The Rt analysis indicated that its value is maximal at the moment of new variant pervasion. As in Earnest [10], we estimated the ratio of Rt numbers during variant circulation to assess the transmissibility of SARS-CoV-2. The Alpha variant was 9% more transmissible than the original strains, Delta was 57% more transmissible than Alpha, and Omicron was 75% more transmissible than Delta. We obtained transmissibility values similar to those previously described for western Europe and America [9,10,15,27]. It is worth noting that, in the early years of the pandemic, there were few sequences that potentially affected the accuracy of determining the period of dominance of certain lines. Obtaining new data for this period will significantly refine Rt values and help determine changes in transmissibility.

Genetic changes in the RBD in Delta and Omicron variants significantly increased the affinity for the ACE2 receptor as L452R, T478K, and F486V mutations emerged [28,29,30,31]. High affinity coupled with a higher viral load in the case of the Delta variant in COVID-19 patients was two orders of magnitude higher than earlier variants and determined the high transmissibility of the virus [32]. The spread of these variants in partially immune populations also contributes to the ability of the aforementioned mutations to evade the neutralising activity of antibodies [33].

We found that the viral load was higher during the spread of Delta, whereas it was significantly lower for Wuhan and Omicron variants (*p* < 0.0001). This complements the previously described data regarding the higher transmissibility of Delta [27,34]. The increased transmissibility of this variant contributed to its spread among the insufficiently immunised population of Moscow.

The virus transmission, as well as other integral indicators, is affected not only by the mutation profile, but also by the level and quality of the population immunity formed as a result of vaccination and/or the COVID-19 contraction, host genetic factors, which may depend on specific human population [35,36], concomitant comorbid diseases [37], the level of certain cytokines in the blood [38], and a number of other factors [39] may play role. It is noteworthy that, the picture of the introduction of new variants of the SARS-CoV-2 virus remains the same regardless of the country, but regional features are also traced. So, in Russia, the dominance of the Wuhan variant and its descendants Alpha, Delta, and Omicron, is noted. At the same time, the Alpha variant did not 50% of the level in the incidence structure (the circulation period is approximately from December 2020 to June 2021), while in the UK, Germany, and a number of other countries, this variant became dominant (according to GISAID data). During this period, a mass vaccination campaign was just beginning in Russia, which once again underlines the possible role of the above factors and the importance of correctly extrapolating the results obtained to other countries and populations of people.

The level of protection against the Delta variant as a result of using the Sputnik V and Sputnik Lite vaccines remained high. This was confirmed by previous studies on virus-neutralising antibodies (VNAs) in which VNAs against Delta were reduced by 2.5-fold [40,41,42]. Our data indicate that during the period of Delta dominance, the CT value in vaccinated patients was higher than that in the primary disease patients, which further indicates the efficacy of the vaccination. In the case of the Omicron variant, no reduction in viral load was observed in vaccinated patients. The composition of the S protein and RBD domain mutations in the Omicron variant indicates the formation of a new virus serotype [43]. This is consistent with data on the reduction in VNAs of sera vaccinated against Omicron by more than 8-fold [44].

Our earlier results for the BA.1, BA.1.1, and BA.2 lines indicate that the efficacy of Sputnik V vaccination in protecting against severe disease remains quite high [45]. However, the continued emergence of new infections and the recurrent occurrence of mutations that increase binding to the ACE2 receptor (L452R and F486V) in later variants of Omicron (BA.4.X and BA.5.X) indicate a significant decrease in vaccine effectiveness, which highlights the need to update the vaccines used in Moscow.

## 4. Materials and Methods

### 4.1. Ethics

We collected swabs routinely from the time of Delta variant entry in different groups of volunteers, including those who were initially infected or vaccinated. For all samples, we evaluated the RT-PCR cycle threshold (Ct) values for SARS-CoV-2 (the lower the Ct, the higher the viral load) and sequenced the complete genome. The data were supplemented with information from the GISAID database and public registries of vaccinated and sick individuals to compile a comprehensive view of the relationships between circulating strains, their virological and epidemiological features, and the effectiveness of vaccination with regard to level immunity.

Written notification was received from all patients in accordance with the order of the Ministry of Health of Russia (21 July 2015 No. 474n). All samples were de-identified prior to their receipt by the research team. The study was approved by the local ethics committee of the Gamaleya National Research Institute of Epidemiology and Microbiology (Protocol No. 14, 29 September 2021). Further sequencing work was carried using Ion Torrent (Thermo Fisher Scientific, Waltham, MA, USA) and Illumina (Illumina, San Diego, CA, USA) technology.

### 4.2. Sample Collection and RT-PCR Testing (Laboratory 1)

Nasopharyngeal swabs were collected from virus transport media (Catalog number G00155, HEM, Russia). Total RNA was extracted using the QIAamp Viral RNA Mini Kit (Qiagen, Germany) and RNA isolation kit to isolate total RNA from animal and bacterial cells, swabs, and viruses on columns (Catalog number RU-250, Biolabmix, Novosibirsk, Russia). Quantitative reverse transcription PCR was conducted using the SARS-CoV-2 FRT RT-PCR kit (Catalog number EA-128, N.F. Gamaleya NRCEM, Moscow, Russia). Specimens with Ct values < 30 were selected for whole-genome sequencing.

### 4.3. Sample Collection and RT-PCR Testing (Laboratory 2)

Nasopharyngeal swabs were collected from virus transport medium (physiological solution or XK-PCR30 transport medium (Jiangsu Xinkang Medical Instrumet Co., Ltd., Taizhou, Jiangsu, China)) or HEM transport medium (Hem, Moscow, Russia). Quantitative reverse transcription PCR was performed using the AmpliPrime SARS-CoV-2 DUO kit (NextBio, Moscow, Russia) according to the manufacturer’s instructions. Specimens with Ct values < 30 were selected for whole-genome sequencing.

### 4.4. Library Preparation and Sequencing (Ion Torrent)

Whole-genome amplification of the SARS-CoV-2 virus genome was performed using the ARTIC primers Itokawa (before 23 August 2021) and V4 (after 24 August 2021) with RT-PCR using BioMaster RT-PCR-Premium (Catalog number RM05-200, Biolabmix, Novosibirsk, Russia). DNA libraries were constructed using the NEBNext Fast DNA Fragmentation and Library Prep Set for Ion Torrent (New England Biolabs, Ipswich, MA, USA), according to the manufacturer’s instructions. DNA sequencing was performed using the Ion 540 Chip and Ion S5XL System or GeneStudio S5 System (Thermo Fisher Scientific, Waltham, MA, USA).

### 4.5. Library Preparation and Sequencing (Illumina)

Libraries were prepared to sequence isolated SARS-CoV-2 RNA according to the following procedure: First, samples were enriched with SARS-CoV-2 genome sequences by reverse transcription and multiplex amplification using specific primers; cDNA synthesis from the viral RNA matrix and subsequent amplification were performed sequentially in a single tube containing a mixture of enzymes for reverse transcription and amplification. Each test sample was subjected to two RT-PCR reactions, which were performed in parallel with two mixes of specific primers for multiplex amplification (manufacturer DiaSystems, Moscow, Russia). The kits used for multiplex amplification of primers contained gene-specific parts from the database of the International Consortium of Researchers ARTIC network (Advancing Real Time Infection Control), an international consortium for infection analysis that offers primers for SARS-CoV-2 genome amplification and now comprises the international standard. Second, the OT-PCR products were re-amplified without intermediate purification using a set of adapter oligonucleotides compatible with the MiSeq sequencing platform (Illumina, San Diego, CA, USA). Sequencing was carried out using the MiSeq 600 cycles v3 reagent kit (Illumina, San Diego, CA, USA) run in 2 × 250 bp reading mode.

### 4.6. NGS Data Analysis (Ion Torrent)

Next-generation sequencing data analysis was performed as previously described [46]. Briefly, the ARTIC primers were trimmed using Cutadapt v3.1 [47]. Reads were trimmed using a quality filter via vsearch v2.17.0, and reads smaller than 100 nt were discarded [48]. The trimmed reads were aligned to SARS-CoV-2 Wuhan-Hu-1 (MN908947.3) using BWA-MEM v0.7.17-r1188 [49]. Variant calling and consensus sequence generation were performed using FreeBayes v1.3.5 [50], bcftools v1.12 [51], and bedtools v2.30.0 [52]. Regions with less than 10-fold coverage were masked. Lineages were assigned with Pangolin v.4.0.6 using pango-data v.1.9.

### 4.7. NGS Data Analysis (Illumina)

The data obtained during bioinformatics analysis were subjected to quality control as follows: The raw reads in FASTQ format from the Illumina MiSeq Dx instrument were first filtered for quality using Trimmomatic v0.39 with a Q20 [53]. Next, data filtered by read quality were aligned to the standard reference Wuhan-Hu-1 comparison genome (MN908947.3) using minimap2 v2.24 [54]. The primer sequences of the multiplex panel for RT-PCR that did not contain genomic information were removed from the aligned reads. Then, searching (colling) for variants was performed using GATK v4.2.6.1 [55]; specifically, mapped reads were compared with the comparison genome to identify variants (differences) of the analysed sample relative to the reference genome if more than five were covered. Based on the variants found, the consensus sequence of the SARS-CoV-2 genome was collected in fasta format using the Bsftools program [51]. Lineages were assigned with Pangolin v.4.0.6 using pango-data v.1.9.

### 4.8. Characterisation of the Genetic Landscape of SARS-CoV-2 Lines

The information on genetic line circulation used in this study was supplemented with data obtained from the GISAID database using the following queries: ‘Location: Europe/Russia/Moscow’ and ‘Location: Europe/Russia/Moscow/Moscow’, ‘Host: Human’, and ‘Complete sequence: Yes’ (Request date 20 August 2022). We re-linearised the resulting 6550 filtered sequences using pangolin v.4.0.6, pango-data v.1.9. The lineage information for the remaining sequences was grouped as follows: Wuhan (B.1 + B.1.X), Alpha (B.1.1.7 + Q.X), Delta (B.1.617.X + AY.X), and Omicron (B.1.1.529 + BA.X). The remaining lines were merged into the ‘Other’ group. The results obtained were combined with metadata, followed by plotting in the R environment using dplyr v1.0.9 and ggplot2 v3.3.5 [56] packages.

### 4.9. Logistic Growth Rates

Logistic growth rates were analysed as previously described [10]. Briefly, the emergence periods for Alpha, Delta, and Omicron variants were defined as the time from the first detection of each genetic line in the database to 90 days afterwards. As a predictor, the number of days since the first detection and genetic line was used as the outcome for binomial logistic regression. The smoothed fitted curves were visualised and evaluated to determine the probability of a sequence belonging to a certain genetic lineage over time. During the analysis, we aimed to include the discovery of new Omicron lines (Figure S2), which made it possible to reveal a faster substitution of BA.1.X + BA.2.X by the BA.5.2 genetic line.

### 4.10. Estimation of Reproductive Number for Different Lines SARS-CoV-2

Given the relatively small number of daily sequences deposited in the GISAID database at the beginning of the pandemic, several assumptions were made. First, we believe that the share of the Wuhan group was dominant from 2020-02 to 2020-11 with over 95–99% representation (Figure 2). Therefore, when calculating the number of cases with the Wuhan variant, we equated it to 100% of the number of initially detected cases. Second, during the transition period of the cocirculation of the Wuhan and Alpha variants, a more aligned number of sequences by day was noted, and the missing values were filled in by interpolation using dplyr v1.0.9. The resulting calculated proportions were converted to the number of cases of infection with a particular line of virus, and a 7-day moving average of infections was used to calculate Rt using the EpiEstim [57] with parameters as described previously [10]: 21-day rolling window, mean serial interval of 5.2 days (2.2 to 8.2 days), and standard deviation of 4 days (2.5 to 5.5 days).

### 4.11. Statistical Analysis

To compare more than two independent groups, we used the Kruskal–Wallis test. If the results were statistically significant, Dunn’s test was used to determine which groups were different. For statistical analysis, we used ggstatsplot v0.9.3 [58] and R v4.1.1.

## 5. Conclusions

The influence of Omicron spectrum variants on the epidemiological situation can be predicted by the analysis of logistic growth curves as well as the viral load in vaccinated individuals. Thus, 50% displacement by the Omicron variant in Moscow occurred 37 days earlier than displacement by the Delta variant of the previously dominant variants. Importantly, displacement occurred against a background of significantly higher levels of vaccination with Sputnik V and Sputnik Lite. Our data suggest that the logistic growth rate and viral load in vaccinated individuals who fell ill with the variant under study can serve as early indicators for selecting candidates for updated vaccines, along with direct assessment of efficacy and assessment of the reduction in viral neutralising activity to the new strains. Cumulative data on the spread of the Omicron variant in Moscow indicate the low efficacy of the established level of population immunity, which highlights the need to optimise the antigenic composition of vaccines used to manage the Omicron virus variant.

## Data Availability

All sequence data are available online (EPI_ISL_1710849-1710866, EPI_ISL_2296111-2296286, EPI_ISL_2296288-2296379, EPI_ISL_4572812, EPI_ISL_5334362-5334371, EPI_ISL_5334374-5334389, EPI_ISL_7211325-7211326, EPI_ISL_7263932-7263933, EPI_ISL_9230058-9230062, EPI_ISL_9230064-9230100, EPI_ISL_10627062, EPI_ISL_11864996-11865125, EPI_ISL_11872910, EPI_ISL_421275, EPI_ISL_454732, EPI_ISL_470896-470904, EPI_ISL_572398, EPI_ISL_872628-872643, EPI_ISL_875515, EPI_ISL_1015362, EPI_ISL_1708507-1708509, EPI_ISL_12225322, EPI_ISL_12748381-12748382, EPI_ISL_13431664-13431687, EPI_ISL_14217225-14217226, EPI_ISL_15327072-15327075, EPI_ISL_15858138-15859137, EPI_ISL_15860713-15860737, EPI_ISL_15860739-15860839, EPI_ISL_15860841-15860991, EPI_ISL_15860993-15861048, EPI_ISL_15862338-15863336, EPI_ISL_15863677-15864655, EPI_ISL_15864802-15865776, EPI_ISL_15865821-15866801, EPI_ISL_15867150-15868141, EPI_ISL_15868158-15869145, EPI_ISL_15869218-15870209, EPI_ISL_15871156-15872140, EPI_ISL_15872157-15873150, EPI_ISL_15873159-15874146, EPI_ISL_15874159-15875146, EPI_ISL_15875638-15876623, EPI_ISL_15876640-15877626, EPI_ISL_15879747-15880730, EPI_ISL_15883551-15884536, EPI_ISL_15884833-15885823, EPI_ISL_15885995-15886980). Inquiries about access to the original clinical data should be directed to the corresponding authors.

## Supplementary Materials

The following supporting information can be downloaded at: https://www.mdpi.com/article/10.3390/ijms232314670/s1.
