# Supplementary material for: Dynamics of SARS-CoV-2 Major Genetic Lineages in Moscow in the Context of Vaccine Prophylaxis
Source: Int J Mol Sci. 2022 Nov 24;23(23):14670. doi: 10.3390/ijms232314670 (PMC9736394; doi:10.3390/ijms232314670)
Supplement: Supplementary file 1 [file ijms-23-14670-s001.zip › ijms-2038843-supplementary captions.docx]

**Figure S1**: Dynamics of changes SARS-CoV-2 major genetic lineages in Moscow.

**Figure S2**: Binomial logistic regression for predicting the probability of sequenced sequences belonging for Alpha, Delta, Omicron including BA.1.x, BA.2.x, BA.5.2, and Omicron other

**Figure S3**: Composition of mutations within the Spike protein of the main genetic variants that dominated in Moscow (Alpha, Beta, Delta, Omicron and endemic variants SARS-CoV-2 in Moscow).
