# Supplementary figures and images for: Dynamics of SARS-CoV-2 Major Genetic Lineages in Moscow in the Context of Vaccine Prophylaxis
Source: Int J Mol Sci. 2022 Nov 24;23(23):14670. doi: 10.3390/ijms232314670 (PMC9736394; doi:10.3390/ijms232314670)

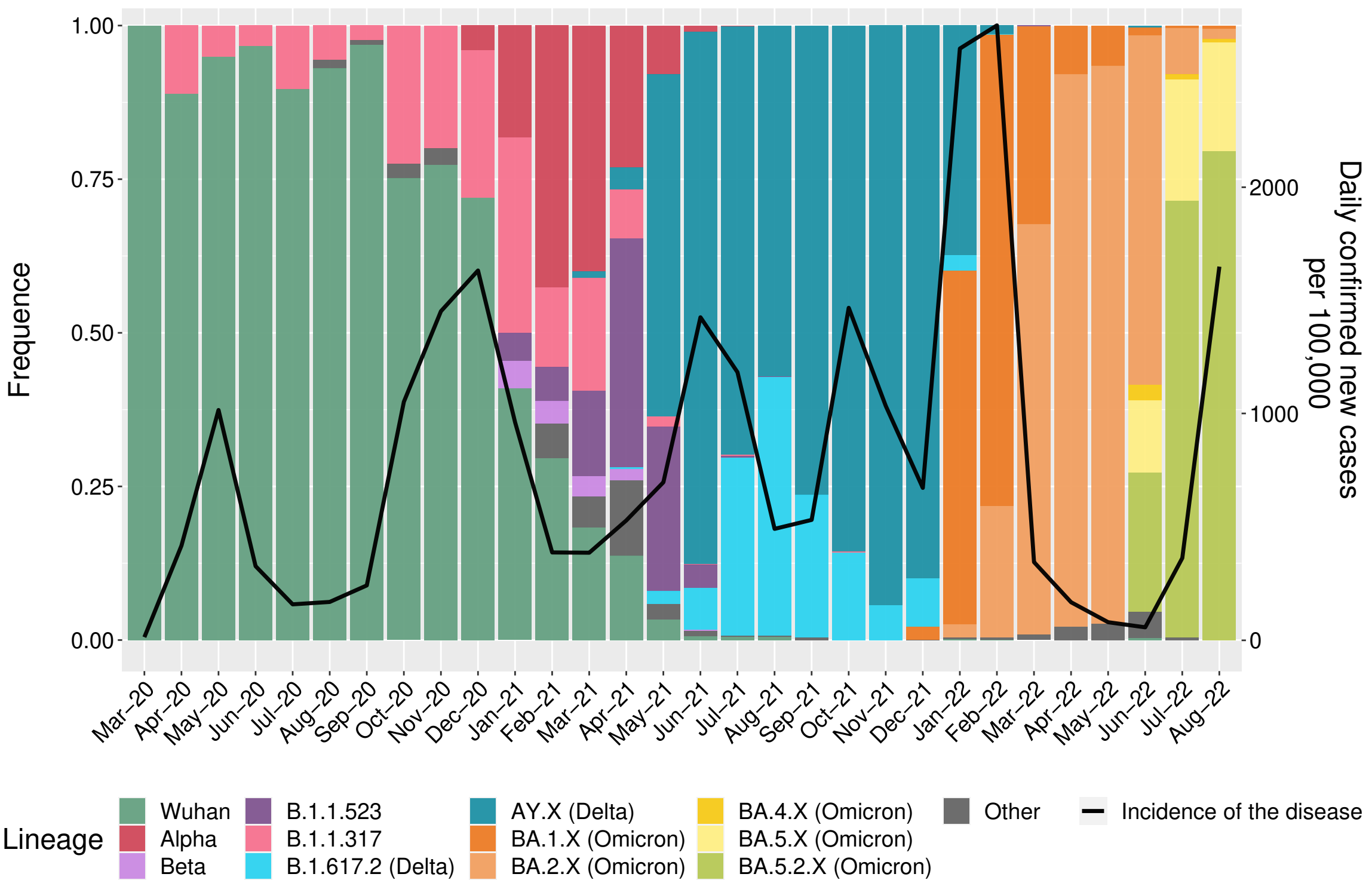

Supplement: Supplementary file 1 [file ijms-23-14670-s001.zip › Figure S1.pdf]

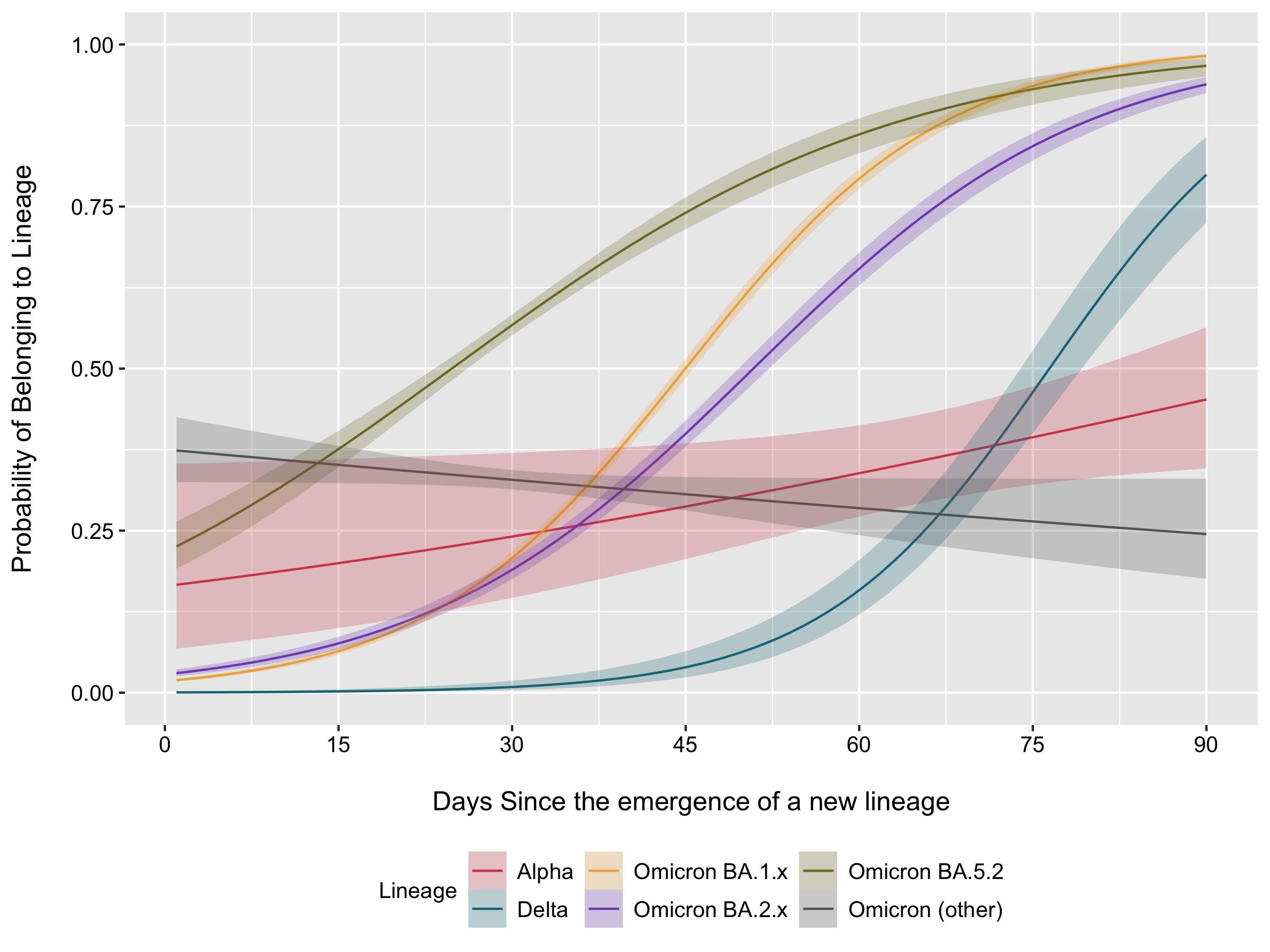

Supplement: Supplementary file 1 [file ijms-23-14670-s001.zip › Figure S2.jpg]

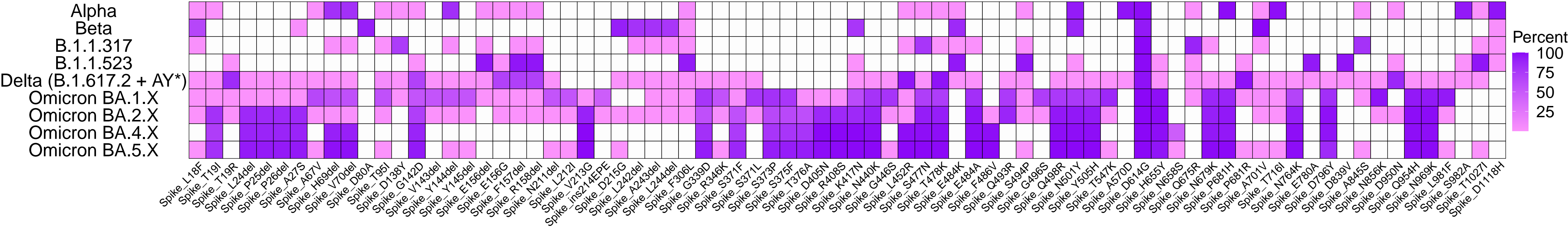

Supplement: Supplementary file 1 [file ijms-23-14670-s001.zip › Figure S3.pdf]
